# The effect of artificial intelligence-supported applications on critical thinking motivation and clinical decision making in nursing students: path analysis

**DOI:** 10.1186/s12909-026-08829-0

**Published:** 2026-03-06

**Authors:** Serap Özdemir, Sümeyye Akçoban, Soner Berşe, Betül Tosun

**Affiliations:** 1 Department of Nursing, Faculty of Health Sciences, Gaziantep University, Gaziantep, Türkiye Turkey; 2 Health Services Department, Kırıkhan Vocational School, Hatay Mustafa Kemal University, Hatay, Türkiye Turkey; 3 Faculty of Nursing, Hacettepe University, Ankara, Türkiye Turkey

**Keywords:** Artificial intelligence, Critical thinking motivation, Clinical decision making, Nursing students, Path analysis

## Abstract

**Background:**

Artificial intelligence (AI)–supported applications are increasingly integrated into nursing education and have the potential to influence students’ cognitive and decision-making processes. Understanding how nursing students’ attitudes toward AI relate to critical thinking motivation and clinical decision-making is essential for designing pedagogically effective AI-enhanced learning environments.

**Purpose:**

This study aimed to examine the relationship between nursing students’ attitudes toward artificial intelligence, critical thinking motivation, and clinical decision-making attitudes using path analysis.

**Materials and methods:**

This cross-sectional quantitative study was conducted between 9 and 20 October 2025 with 419 nursing students who had clinical experience at a state university in the southeastern region of Türkiye. In the study, it was census of eligible students’ method and single center. Data were collected online using the Student Demographic Information Form, the Artificial Intelligence Attitude Scale for Nurses, the Critical Thinking Motivation Scale, and the Clinical Decision Making in Nursing Scale. Descriptive statistics, correlation analysis, multiple regression, and path analysis were performed using SPSS 26.0 and R 4.5.1.

**Results:**

Students reported moderately positive attitudes toward artificial intelligence (mean = 3.26 ± 0.38), high levels of critical thinking motivation (mean = 4.31 ± 0.93), and moderately high clinical decision-making attitudes (mean = 3.26 ± 0.33). Attitudes toward AI were positively associated with critical thinking motivation; however, no significant direct association with clinical decision-making attitudes was observed. Critical thinking motivation was a strong predictor of clinical decision-making attitudes and fully mediated the relationship between AI attitudes and clinical decision making. The path model explained 24% (β = 0.488) of the variance in critical thinking motivation and 49% (β = 0.700) of the variance in clinical decision-making attitudes. The standardized indirect effect of AI attitudes on clinical decision-making through critical thinking motivation was significant (β = 0.341, 95% CI [0.232, 0.355]).

**Conclusion:**

Positive attitudes toward artificial intelligence were associated with stronger critical thinking motivation among nursing students, which in turn was associated with higher clinical decision-making attitudes. Critical thinking motivation can mediate nursing students’ delivery of quality care services. AI-supported educational applications may be most effective when designed to explicitly promote critical thinking within structured and pedagogically sound learning environments.

**Clinical trial number:**

Not applicable.

## Contributions to the Literature

- This study provides one of the first path analysis models demonstrating that nursing students’ attitudes toward artificial intelligence influence clinical decision-making indirectly through critical thinking motivation.
- The findings highlight critical thinking motivation as a key cognitive mechanism linking AI-related attitudes to clinical decision-making, an association that has been insufficiently examined in previous nursing education research.
- The results offer practical guidance for nurse educators and curriculum developers by emphasizing that AI-supported applications should be intentionally structured to cultivate critical thinking (e.g., through reflective prompts or delayed AI feedback) to enhance clinical decision-making competence.

## Introduction

In recent years, artificial intelligence (AI) technologies have been rapidly advancing in the healthcare field and are being used in many areas such as clinical decision support systems, imaging, patient monitoring, document management, and communication. In the context of nursing education, AI, particularly large language models (LLMs), is evolving into structured case analyses, problem-based learning environments, and virtual patient simulations, allowing them to apply their theoretical knowledge in a clinical context, innovative tools that support students’ critical thinking and clinical reasoning skills [1–5].

However, the literature also contains initial evidence that problem-based learning (PBL) methods with LLM significantly improve students’ critical thinking skills. For example, students in LLM-integrated PBL areas showed significant improvement in critical thinking tests compared to traditional PBL [6]. Additionally, AI-supported case analyses have been observed to improve case management performance and learning satisfaction, while also positively impacting clinical reasoning skills [3]. In addition, a chatbot-based training program developed in South Korea has been shown to significantly improve nursing students’ clinical reasoning skills, self-confidence, and satisfaction with their education. Such AI-based tools enable students to have safe learning experiences in either synchronous or asynchronous formats. Thanks to the instant feedback feature, clinical decision-making processes develop more systematically and confidently [7].

However, AI-led teaching models in patient simulations supported by virtual reality (VR) positively affect students’ communication, clinical reasoning, and confidence levels [8]. AI-supported case analyses and simulation environments have a positive impact on both case management performance and individual decision-making processes. For example, interactive virtual simulations allow students to experience the steps of the decision-making process (information gathering, problem identification, intervention selection) while providing immediate feedback, thereby developing the ability to make confident and informed clinical decisions [9]. Recently there has been the gap in mediation studies linking AI attitudes to clinical decision-making through critical thinking. It is believed that the relationship between these concepts needs further explanation.

## Background

Literature findings indicate that research examining the impact of artificial intelligence-supported learning applications on nursing students’ critical thinking and clinical decision-making skills is still limited [10–13]. Recent studies have shown that artificial intelligence-supported educational applications make meaningful contributions to nursing students’ learning experiences. Harder et al. (2025) reported that an artificial intelligence-supported virtual reality (VR) application increased students’ clinical decision-making levels [10]. Similarly, Akutay et al. (2024) stated that AI-based case analyses improved student case management performance and increased learning satisfaction [11]. In another study evaluating ChatGPT-assisted learning, it was found that this method significantly enhances students’ clinical reasoning skills compared to traditional text-based learning [12]. Additionally, Benfatah et al. (2024) reported that AI-supported instruction significantly increased nursing students’ critical thinking levels [13].

However, the literature also contains findings that AI-supported applications do not always provide the expected level of learning outcomes in nursing education. Ayed et al. (2025) in the study highlighted the importance of AI-supported applications in enhancing emotional intelligence and critical thinking, as well as cognitive motivation, among nursing students [14]. Ayed (2025) examining the relationship between emotional intelligence and clinical decision-making among nurses in neonatal intensive care units, the researchers reported that the nurses exhibited both high emotional intelligence and strong clinical decision-making skills [15].

Glauberman et al. (2023) stated that artificial intelligence offers significant opportunities in nursing education, but the full potential of these technologies has not been realized due to pedagogical limitations in application, ethical concerns, and the readiness levels of teaching staff. The study emphasized that there is still a lack of strong empirical evidence that AI-based systems directly improve critical thinking and clinical reasoning skills [16]. In their study, Khlaif et al. (2025) noted that while students’ perceptions of generative artificial intelligence (e.g., ChatGPT)-based learning experiences were positive, these tools could limit deep learning, original thinking, and cognitive engagement [17]. These findings indicate that AI-supported learning environments can lead to superficial learning when not carefully structured, highlighting the need for further contributions to the literature on this topic.

In summary, studies comprehensively examining the impact of artificial intelligence-supported learning applications on critical thinking and clinical decision-making skills in nursing students are limited. Most studies evaluate tools or interventions; fewer examine how students’ AI attitudes relate to decision-making through motivation mechanisms. In this context, this research aims to fill the gap in the literature by examining these effects using a path analysis approach.

### Research Hypotheses

- H1: Nursing students’ attitudes toward artificial intelligence are positively associated with their critical thinking motivation.
- H2: Nursing students’ attitudes toward artificial intelligence are positively associated with their clinical decision-making attitudes.
- H3: Nursing students' critical thinking motivation plays a mediating role in the relationship between AI-supported applications and their clinical decision-making competence.

## Materials and methods

### Study design

This study was conducted based on a cross-sectional and quantitative research design aimed at examining directional relationships. The study tested the effect of artificial intelligence-supported applications on nursing students’ critical thinking skills and clinical decision-making competencies using path analysis (*structural equation modeling*).

### Study location and time

The research was conducted at a state university in the Southeastern Anatolia region of Turkey between October 9 and October 20, 2025.

### Study population and sample

The population of this study consists of a total of 700 students enrolled in the nursing department of a state university in the Southeastern Anatolia Region of Turkey during the Fall semester of 2025–2026. The sample of the study consisted of second, third-, and fourth-year students (*n* = 419) who had clinical practice experience. Transfer and exchange students are subject to the nursing department curriculum and therefore have equal responsibilities in all lessons and practical applications like other students. For this reason, they were included in the sample group. First-year students were excluded from the study because they had not yet begun clinical practice and therefore did not participate sufficiently in critical thinking and clinical decision-making processes.

In the study, it was census of eligible students’ method was used to increase the representativeness of the population, and the aim was to include all students with clinical experience in the research. This approach aimed to strengthen the generalizability and representativeness of the findings. Sample adequacy was assessed using a post hoc G*Power analysis based on the R² values obtained in the study. The analysis found an effect size of f² = 0.316–0.96 and 1–β > 0.999 for the structural model, confirming that the sample size was statistically adequate.

*Inclusion Criteria*: Proficient in reading and writing Turkish, enrolled in the 2nd, 3rd, or 4th year of the nursing program, and actively pursuing their studies. Nursing students who voluntarily agreed to participate in the study.

## Results

A total of 419 nursing students participated in the study, with an average age of 20.73 ± 2.24 years. The majority of students were female (73.5%, *n* = 308). In terms of class level distribution, the highest rate was observed among third-year students (*n* = 228) at 54.4%; this was followed by second-year students (*n* = 145) at 34.6% and fourth-year students (*n* = 46) at 11.0%. In terms of income status, the highest rate was found among students with equal income and expenditure (*n* = 264) at 63.0%.

Most participants (83.8%, *n* = 351) reported using artificial intelligence applications in nursing education. Regarding the impact of artificial intelligence applications on clinical decision-making, 59.9% of students responded “it has an impact,” 37.0% responded “partially,” and 3.1% responded “no.” The most frequently used resource in the clinical decision-making process was the internet/smartphone (34.4%, *n* = 144), followed by lecture notes (24.6%), academic resources (20.5%), artificial intelligence applications (17.7%), and peers (2.9%) (Table 1).

No statistically significant gender differences were observed in AI attitude or critical thinking motivation (*p* > 0.05). However, female students demonstrated significantly higher clinical decision-making scores than male students (t = 2.57, *p* = 0.010). Grade level, however, had a significant effect on AI attitude (F = 6.80, *p* = 0.001) and critical thinking motivation (F = 3.32, *p* = 0.037). Post-hoc comparisons indicated that second-year students demonstrated higher AI attitude scores than fourth-year students, while third-year students scored higher than fourth-year students in critical thinking motivation. Clinical decision-making did not differ significantly by grade level (F = 0.83, *p* = 0.433).

Income status emerged as a significant determinant across all three constructs (*p* < 0.01). Students whose income was equivalent to their expenses reported higher mean scores in AI attitude, critical thinking motivation, and clinical decision-making competence than those whose income was below their expenses.

Students who had previously used AI applications in their nursing education exhibited significantly higher clinical decision-making scores (t = 2.76, *p* = 0.006). Similarly, students who perceived AI as beneficial for clinical decision-making achieved higher clinical decision-making scores compared to those who perceived little or no benefit (F = 22.28, *p* < 0.001).

With regard to the primary sources of information used in clinical decision-making, students who relied on instructors demonstrated significantly higher clinical decision-making scores than those who depended on the internet or peers (F = 4.59, *p* = 0.001). Moreover, students who reported consistently approaching clinical situations from multiple perspectives and discussing possible outcomes before making a decision obtained significantly higher mean scores across all three scales (*p* < 0.01) (Table 1).

**Table 1:** Comparison of Mean Scores by Sociodemographic and AI-Related Variables (*n* = 419)

Variables Age 20.73 ± 2.24	*n*	AI Attitude (Mean ± SD)	Critical Thinking Motivation (Mean ± SD)	Clinical Decision-Making (Mean ± SD)
Gender				
Female	308	3.25 ± 0.38	4.34 ± 0.90	3.29 ± 0.33
Male	111	3.28 ± 0.38	4.25 ± 1.01	3.20 ± 0.31
* t / p*		*t* = − 0.67, *p* = 0.502	*t* = 0.86, *p* = 0.391	***t*** **= 2.57**, ***p*** **= 0.010**
**Grade Level**				
2nd year	145	3.27 ± 0.33	4.27 ± 0.98	3.24 ± 0.40
3rd year	228	3.29 ± 0.32	4.39 ± 0.90	3.28 ± 0.38
4th year	46	3.10 ± 0.21	4.02 ± 0.87	3.21 ± 0.28
* F / p*		***F*** **= 6.80**, ***p*** **= 0.001**	***F*** **= 3.32**, ***p*** **= 0.037**	*F* = 0.83, *p* = 0.433
* Post-hoc*		**2nd>4th** **3rd >4th**	**3rd > 4th**	
**Income Status**				
Below expenses	117	3.19 ± 0.28	4.09 ± 0.90	3.14 ± 0.38
Equal to expenses	264	3.30 ± 0.33	4.43 ± 0.88	3.32 ± 0.36
Above expenses	38	3.28 ± 0.39	4.17 ± 1.25	3.23 ± 0.48
* F / p*		***F*** **= 5.01**, ***p*** **= 0.007**	***F*** **= 5.89**, ***p*** **= 0.003**	***F*** **= 10.02**, ***p*** **< 0.001**
* Post-hoc*		Equal > Below	Equal > Below	Equal > Below
**Use of AI in Nursing Education**				
Yes	351	3.28 ± 0.32	4.35 ± 0.91	3.28 ± 0.38
No	68	3.20 ± 0.33	4.14 ± 1.03	3.15 ± 0.39
* t / p*		*t* = 1.80, *p* = 0.073	*t* = 1.67, *p* = 0.096	***t*** **= 2.76**, ***p*** **= 0.006**
**Perceived impact of AI applications on clinical decision-making**				
Yes	251	3.28 ± 0.33	4.36 ± 0.94	3.35 ± 0.39
Partly	155	3.24 ± 0.32	4.26 ± 0.86	3.15 ± 0.31
No	13	3.16 ± 0.34	4.08 ± 1.42	2.86 ± 0.48
* F / p*		*F* = 1.58, *p* = 0.206	*F* = 0.98, *p* = 0.377	***F*** **= 22.28**, ***p*** **< 0.001**
* Post-hoc*		—	—	Yes > Partly > No
**Main source used in clinical decision-making**				
Lecture notes	103	3.19 ± 0.35	4.33 ± 0.92	3.19 ± 0.35
Academic	86	3.23 ± 0.41	4.47 ± 0.94	3.36 ± 0.38
Internet / smartphone	144	3.30 ± 0.37	4.23 ± 0.94	3.21 ± 0.29
AI applications	74	3.35 ± 0.39	4.37 ± 0.87	3.27 ± 0.29
Peers (friends)	12	3.03 ± 0.33	3.72 ± 1.08	3.03 ± 0.23
* F / p*		***F*** **= 3.51**, ***p*** **= 0.008**	*F* = 2.21, *p* = 0.067	***F*** **= 4.59**, ***p*** **= 0.001**
* Post-hoc*		—	—	Lecture notes > Internet / smartphone Lecture notes > Peers
**Tendency to approach clinical situations from multiple perspectives**				
Yes	190	3.33 ± 0.44	4.64 ± 0.91	3.38 ± 0.35
Partly	217	3.21 ± 0.31	4.06 ± 0.82	3.17 ± 0.27
No	12	3.11 ± 0.53	3.71 ± 1.34	3.13 ± 0.30
* F / p*		***F*** **= 6.75**, ***p*** **= 0.001**	***F*** **= 25.23**, ***p*** **< 0.001**	***F*** **= 23.78**, ***p*** **< 0.001**
* Post-hoc*		**Yes > Partly**	**Yes > Partly > No**	**Yes > Partly > No**
**Discuss possible outcomes before making a clinical decision**				
Yes	191	3.39 ± 0.35	4.66 ± 0.92	3.33 ± 0.44
Partly	209	3.15 ± 0.25	4.05 ± 0.83	3.22 ± 0.31
No	19	3.17 ± 0.28	3.80 ± 1.05	3.11 ± 0.40
* F / p*		***F*** **= 32.35**, ***p*** **< 0.001**	***F*** **= 27.37**, ***p*** **< 0.001**	***F*** **= 5.81**, ***p*** **= 0.003**
* Post-hoc*		**Yes > Partly & No**	**Yes > Partly > No**	**Yes > Partly > No**

The *Artificial Intelligence Attitude Scale for Nurses (AIAS-N)* demonstrated good internal consistency (α = 0.847), with an overall mean item score of 3.26 ± 0.38. Among its subscales, mean item scores ranged from 2.60 to 3.52, and internal consistency coefficients varied between 0.802 and 0.887, indicating satisfactory reliability.

The *Critical Thinking Motivation Scale (CTMS)* showed excellent internal consistency (α = 0.971) with an overall mean item score of 4.31 ± 0.93, suggesting that students generally reported a high level of motivation for critical thinking.

The *Clinical Decision Making in Nursing Scale (CDMNS)* also demonstrated strong reliability (α = 0.831), with a mean item score of 3.26 ± 0.33. Across its four subscales, mean item scores ranged from 2.86 to 3.61, and α values between 0.674 and 0.911, indicating acceptable to excellent internal consistency (Table 2).

**Table 2:** Reliability and Descriptive Statistics of the Study Scales (*n* = 419)

Scale / Subscale	*n* (items)	Cronbach’s α	Mean (X̄)	SD	Min	Max
**Artificial Intelligence Attitude Scale for Nurses (Total)**	32	0.847	3.26	0.38	46	152
Nursing Care	14	0.802	3.43	0.50	24	68
Organization	6	0.887	3.52	0.68	6	30
Ethics	6	0.880	2.60	0.70	6	30
AI Readiness	6	0.863	3.28	0.69	6	30
**Critical Thinking Motivation Scale (Total)**	19	0.971	4.31	0.93	19	114
**Clinical Decision Making in Nursing Scale (Total)**	40	0.831	3.26	0.33	104	172
1. Searching for Alternatives or Options	10	0.911	3.61	0.66	10	50
2. Canvassing Objectives and Values	10	0.894	3.58	0.63	10	50
3. Evaluating Consequences	10	0.674	3.01	0.49	17	44
4. Information Search and Unbiased Assimilation	10	0.832	2.86	0.41	16	42

The hypothesized mediation model included three observed variables (AI Attitude, Critical Thinking Motivation, and Clinical Decision-Making) and three structural paths. This specification constitutes a just-identified (saturated) model with zero degrees of freedom (df = 0). As saturated models perfectly reproduce the observed variance–covariance matrix, conventional goodness-of-fit indices (e.g., CFI, RMSEA, SRMR) are not applicable [18]. Model adequacy was therefore evaluated based on the significance of individual path coefficients, the proportion of variance explained (R²), and bias-corrected bootstrap 95% confidence intervals for the indirect effect (Table 3).

**Table 3:** Direct, Indirect, and Total Effects in the Mediation Model of Critical Thinking Motivation between AI Attitude and Clinical Decision-Making (*n* = 419)

Path	Label	B	SEboot	z	*p*	95% CI (B)	β (Standardized)
**AI Attitude → Critical Thinking Motivation**	a	1.188	0.113	10.475	**< 0.001**	[0.970, 1.415]	0.488
**AI Attitude → Clinical Decision Making**	c′	−0.031	0.037	−0.833	0.405	[− 0.102, 0.042]	−0.036
**Critical Thinking Motivation → Clinical Decision Making**	b	0.244	0.015	16.470	**< 0.001**	[0.215, 0.274]	0.700
**Indirect effect (a × b)**	ind	0.290	0.032	9.191	**< 0.001**	[0.232, 0.355]	0.341
**Total effect (c′ + a × b)**	tot	0.259	0.042	6.152	**< 0.001**	[0.178, 0.342]	0.305

The results indicated that AI Attitude had a significant positive effect on Critical Thinking Motivation (B = 1.188, SE = 0.113, z = 10.475, *p* < 0.001, β = 0.488). In turn, Critical Thinking Motivation significantly predicted Clinical Decision Making (B = 0.244, SE = 0.015, z = 16.470, *p* < 0.001, β = 0.700). However, the direct effect of AI Attitude on Clinical Decision Making was nonsignificant (B = − 0.031, SE = 0.037, z = − 0.833, *p* = 0.405, β = −0.036).

**Fig. 1:**
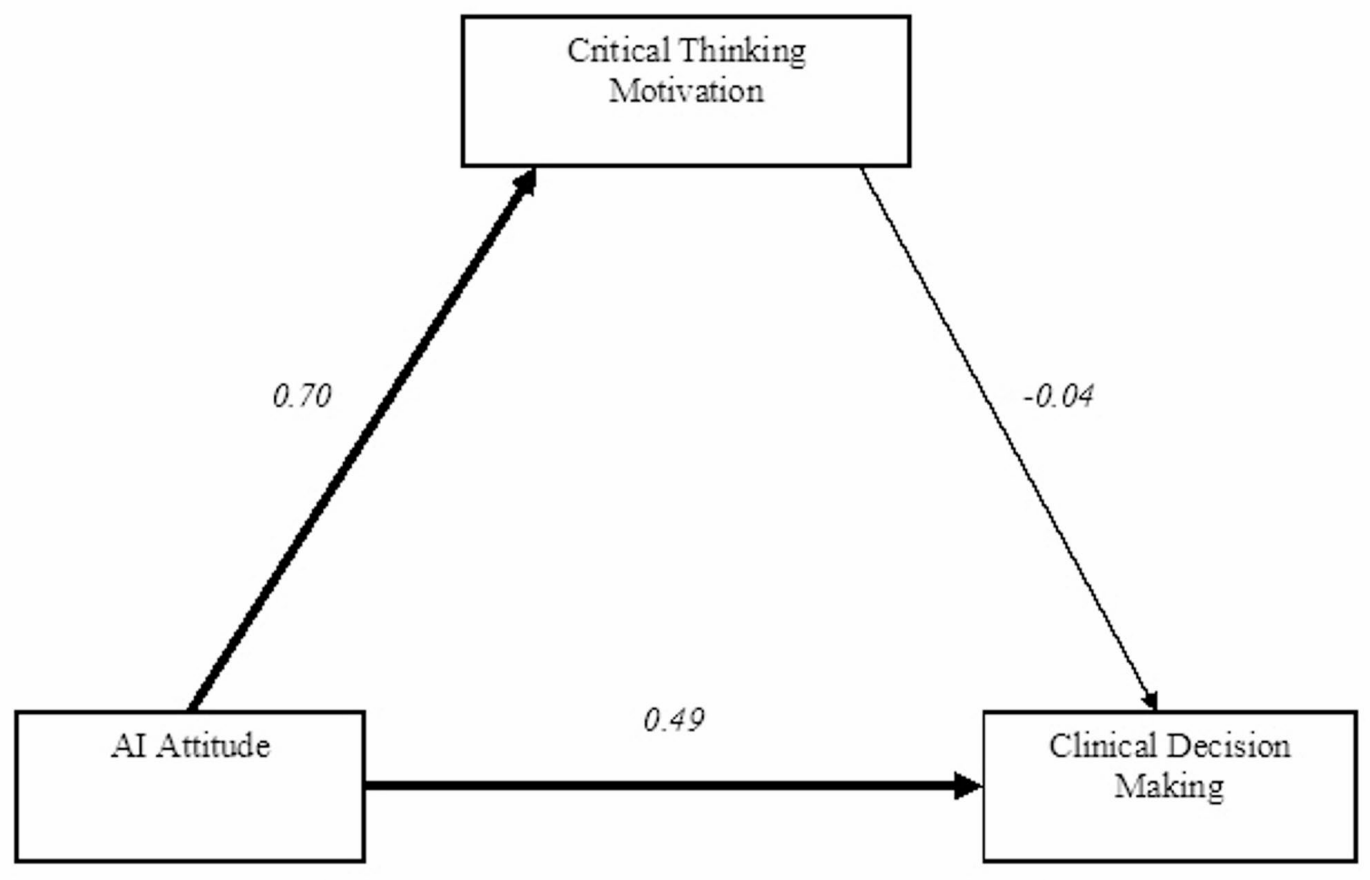
Path diagram

The indirect effect of AI Attitude on Clinical Decision Making through Critical Thinking Motivation was significant (B = 0.290, SE = 0.032, z = 9.191, *p* < 0.001, 95% CI [0.232, 0.355], β = 0.341), indicating a meaningful mediation pathway. The total effect (direct + indirect) was also significant (B = 0.259, SE = 0.042, z = 6.152, *p* < 0.001, β = 0.305) (Table 3; Fig. 1).

The model explained 24% of the variance in critical thinking motivation (R² = 0.24) and 49% of the variance in clinical decision-making skills (R² = 0.49). This finding indicates that nursing students’ attitudes toward artificial intelligence are a significant determinant in their cognitive and decision-making processes (Table 3) (Fig. 1).

## Discussion

In recent years, the development of artificial intelligence technologies has also affected healthcare services. One of the important factors in holistic nursing care is critical thinking, which allows for the accurate assessment of the positive and negative outcomes of the practice [2, 8]. Another is being able to make quick and safe clinical decisions that will affect the quality of care. Nursing education has a practical training model. Students’ ability to combine theoretical knowledge and practice is an important criterion in developing their professional skills [7, 9].

This study found that participants generally had a positive attitude toward artificial intelligence. Similarly, Akutay et al. (2024) reported that nursing students expressed a high level of satisfaction with AI-focused case studies [11]. Benfatah et al. (2024) reported that students in AI-supported education were more confident, knowledgeable, skilled, and satisfied compared to other groups [13]. In contrast, Ayed et al. (2025) in the study reported a contrast between nursing students’ positive attitudes and their anxiety about artificial intelligence [14].However, this study found that the ethical sub-dimension of attitudes toward artificial intelligence was at a moderate level. This finding is consistent with the results reported by Shin et al. (2024), which showed that traditional training modules are more effective in implementing ethical standards [12]. In general, it is thought that artificial intelligence applications support the learning process in nursing education but have not yet fully met expectations in terms of ethical approach. Therefore, it is predicted that hybrid models integrating artificial intelligence with traditional methods in nursing education may be more effective, particularly in terms of ethics. And additionally, it is believed that artificial intelligence alone cannot improve the decision-making process without the involvement of critical thinking.

This study found that participants’ motivation for critical thinking was quite high. Similarly, Benfatah et al. (2024) reported that nursing students were more willing to participate in applications and that their critical thinking levels had increased [13]. Kejingyun & Mingjun (2025), on the other hand, reported in their study that nursing students have high critical thinking skills [6]. It is considered a professional requirement for nursing students to use their critical thinking skills in evaluating the effectiveness of healthcare practices and in predicting possible positive or negative outcomes.

Our study found that participants’ attitudes toward clinical decision-making were significantly high. Similarly, in a study by Harder et al. (2025) comparing artificial intelligence and virtual reality simulation applications, it was reported that nursing students made decisions faster thanks to the use of artificial intelligence [10]. In their study, Medel et al. (2025) reported that effective methods used in clinical decision-making by nursing students positively influenced confident and conscious clinical decision-making processes [9]. Shin et al. (2024) reported that nursing students using artificial intelligence demonstrated more effective time management in clinical decision-making [12]. It can be said that nursing students’ attitudes toward clinical decision-making have increased due to the impact of developing technology, and that students’ use of technological approaches in research and development processes has contributed to this increase.

It was found that third-year female nursing students with equal income levels had higher attitudes toward artificial intelligence. Saydamlı et al. (2025) reported that female nursing students showed a more positive attitude toward artificial intelligence, and that third-year nursing students with income levels equal to their expenses had higher attitudes toward artificial intelligence compared to first- and second-year students [19]. Sözen et al. (2024) reported that third-year nursing students and male students had a higher attitude toward artificial intelligence [20]. The higher artificial intelligence attitudes of nursing students in their third year are consistent with previous studies. This can be explained by the well-developed level of their professional knowledge and skills. The gender difference can be interpreted as being influenced by family structure and different sociocultural characteristics.

According to the findings of this study, nursing students’ attitudes toward artificial intelligence positively influenced their critical thinking motivation, but no direct effect was found on their clinical decision-making attitudes. In contrast, critical thinking motivation significantly increased clinical decision-making attitudes. It was determined that the artificial intelligence attitude model explained one-quarter of the variance in critical thinking motivation and nearly half of the variance in clinical decision-making attitudes. These findings are largely consistent with current research in the literature. Harder et al. (2025) found that AI-based applications improved students’ clinical decision-making skills, Akutay et al. (2024) found that they increased case management performance and learning satisfaction, Shin et al. (2024) found that AI-supported learning strengthened the level of clinical decision-making, and Benfatah et al. (2024) report that AI-supported education meaningfully develops nursing students’ critical thinking skills [10–13]. When these results are evaluated together, it can be said that AI-based learning environments strengthen students’ critical thinking motivation and that this motivation plays an indirect but significant role in clinical decision-making processes. Therefore, it is seen that AI-supported approaches in nursing education support clinical decision-making skills, particularly through an intermediate variable that strengthens critical thinking.

### Limitations

This study has several limitations that should be considered when interpreting the findings. First, the sample was drawn from a single state university in Türkiye, which may limit the generalizability of the results to nursing students in different geographic regions, institutional contexts, or educational systems. Second, data were collected through self-reported online questionnaires, which may be subject to response bias, including social desirability bias or inattentive responding.

Third, although path analysis was employed to examine directional relationships among variables, the cross-sectional design precludes definitive causal inferences. Therefore, the observed associations should be interpreted as relational rather than causal. Fourth, the study relied exclusively on quantitative data; qualitative insights into students’ experiences with AI-supported learning environments were not explored, limiting the depth of contextual interpretation. Finally, potentially influential factors such as instructional design quality, faculty competence in AI integration, institutional digital infrastructure, and ethical guidance regarding AI use were not examined and may also affect critical thinking motivation and clinical decision-making attitudes.

Fourth, The AI Attitude Scale for Nurses (AIASN) Cronbach’s α in this study was 0.847, which is acceptable, but the subscale reliabilities vary.

Fifth, Cross-sectional design can preventing causal inference and may introduce the potential for common methodological bias stemming from self-report measures.

## Conclusion and Future Directions

This study demonstrated that nursing students exhibited generally positive attitudes toward artificial intelligence, high levels of critical thinking motivation, and moderately high clinical decision-making attitudes. Attitudes toward artificial intelligence were positively associated with critical thinking motivation; however, no direct association with clinical decision-making attitudes was identified. Instead, critical thinking motivation played a full mediating role, indicating that AI-related attitudes enhance clinical decision-making primarily by strengthening students’ motivation to think critically.

Students who actively used AI applications, approached clinical situations from multiple perspectives, discussed potential outcomes before making decisions, and perceived AI as beneficial for clinical decision-making reported higher levels of critical thinking motivation and clinical decision-making attitudes. These findings highlight critical thinking motivation as a central cognitive mechanism linking AI-supported learning to clinical decision-making competence. We are suggestion for mixed-methods follow-up studies to explore student experiences qualitatively.

Based on the findings, the following recommendations are proposed:

### Integration of AI-Supported Learning

- Incorporating AI-supported case analyses, simulations, and decision-support tools into undergraduate nursing curricula in a structured and pedagogically guided manner.
- Ensuring that AI applications are used as complementary tools alongside traditional teaching methods rather than as standalone solutions.

### Strengthening Critical Thinking and Ethical Awareness

- Designing learning activities that explicitly promote critical thinking, reflection, and analytical reasoning within AI-supported environments.
- Integrating ethical frameworks and guidance to support responsible and conscious use of AI technologies in nursing education.

### Research and Educational Development

- Conducting multi-center studies with larger and more diverse samples to improve generalizability.
- Employing mixed-methods and longitudinal designs to explore students’ experiences with AI-supported education and to evaluate long-term effects on professional competence and clinical performance.

Implementing these strategies may contribute to the development of more reflective, analytically skilled, and confident nursing graduates, thereby supporting safer, more effective, and evidence-based patient care in increasingly technology-integrated healthcare systems.

### Data collection tools

#### Student Identification Information Form

The form developed by researchers includes statements assessing students’ age, gender, grade level, use of artificial intelligence applications, clinical decision-making, and critical thinking.

### Artificial Intelligence Attitude Scale for Nurses (AIASN)

The scale was developed by Ölçek, Yıldırım & Karaman (2025) to assess nurses’ attitudes toward artificial intelligence [21]. The scale consists of a total of 32 items and 4 subdimensions. The subdimensions are listed as follows: Items 1–14 cover Nursing Care, items 15–20 cover Organization, items 21–26 cover Ethics, and items 27–32 cover Artificial Intelligence Readiness. The scale is scored on a 5-point Likert scale, with “1 = Strongly disagree, 2 = Disagree, 3 = Undecided, 4 = Agree, 5 = Strongly agree.” The scales scores were minimum 32 and maximum 160. A high score on the scale indicates that nurses have a positive attitude towards artificial intelligence. Items 4, 13, and 14 in the Nursing Care sub-dimension and all items in the Ethics sub-dimension are reverse-coded. The overall Cronbach Alpha coefficient of the scale was found to be 0.925. In the subscales, Cronbach’s Alpha values range from 0.91 to 0.96. In this study, the Cronbach Alpha value was found to be 0.847.

### Critical thinking motivation scale

The scale was first developed by Valenzuela, Nieto, and Saiz [22] and consists of 19 items; items are rated on a 6-point Likert scale (1 = Strongly disagree, 6 = Strongly agree). The scales scores were minimum 19 and maximum 114. Higher scores indicate higher critical thinking motivation. The Turkish adaptation and psychometric validity–reliability study was conducted by Dönmez and Kaya [23]. The internal consistency coefficients on the total scale are α = 0.90, and the model–data fit indices are acceptable. In this study, the total internal consistency of the scale was determined to be α = 0.971.

### Clinical decision-making scale in nursing

The scale developed by Jenkins aims to describe each behavior exhibited by nursing students when providing care to patients in various situations [24], The validity and reliability of the Turkish version have been established by Durmaz-Edeer and Sarıkaya [25]. The scale consists of 40 items and four subscales, using a 5-point Likert-type (1 = never, 5 = always). The subscales are, in order, “Researching options and ideas,” “Investigating goals and values,” “Evaluating outcomes,” and “Researching information and adopting new information impartially.” Eighteen items on the scale are reverse-scored, and the total score ranges from 40 to 200, with scores ranging from 10 to 50 for each subscale. The scale does not have a cutoff point. A high score on the scale indicates high decision-making ability, while a low score indicates low decision-making ability. The Cronbach’s alpha reliability coefficient of the nursing clinical decision-making scale adapted into Turkish was found to be 0.78. In this study, the Cronbach’s alpha value was found to be 0.831.

### Data collection

In this study, data were collected through an online survey created by researchers using the Google Forms platform. Before the data collection process, the student representatives of the 2nd, 3rd, and 4th-year nursing departments were contacted and informed about the purpose of the study, that participation was entirely voluntary, that the information collected would be kept confidential, and that it would be used solely for scientific purposes. The survey link was then shared with class representatives and communicated to students via student WhatsApp groups. To prevent duplicate entries, a single access option per device has been added to the research link. It took participants approximately 10–15 min to complete the survey. No personal identification information was requested during the survey, and all responses were stored in a secure digital environment accessible only to the research team. Control questions were integrated into the survey questions to assess the accuracy and attention level of the participants’ responses. This approach was adopted to increase participant confidence, reduce bias in responses, and ensure the reliability of the data obtained. The forms were set up so that each participant could complete the survey only once.

### Ethical considerations

Before initiating the study, ethical approval was obtained from the Non-Interventional Research Ethics Committee of a state university (Date: 17.09.2025; Meeting number: 12; Decision No: 31). In addition, the necessary institutional permission was obtained from the nursing department of the faculty of health sciences where the research was conducted (Number: E-50581566-900-729455). The data collection form was prepared online and included information at the beginning of the form about the purpose of the study, participants’ right to withdraw at any time, and assurance of confidentiality. Students’ voluntary participation and informed consent were obtained through the option “I voluntarily agree to participate in the study” in the first item of the questionnaire. The research was conducted in accordance with the principles of the Declaration of Helsinki. Permission to use the scales was obtained from the original authors via email prior to data collection.

### Data analysis

The data obtained in this study were analyzed using the SPSS 26.0 software package and R (version 4.5.1). Before analysis, data integrity, missing values, and outliers were checked. For continuous variables, the normality assumption was evaluated based on the criterion that skewness and kurtosis coefficients fall within the range of -1.5 to + 1.5 [26]. Descriptive data for participants are summarized using frequency, percentage, mean, and standard deviation. The Tukey HSD test was used after analysis of variance to determine differences between groups. The relationships between students’ attitudes toward artificial intelligence, clinical decision-making levels, and critical thinking motivation were examined using Pearson correlation analysis. Multiple linear regression analysis was performed to determine the factors predicting artificial intelligence readiness. In addition, path analysis was applied to test the direct and indirect relationships between artificial intelligence attitude, clinical decision-making, and critical thinking.

## Data Availability

The datasets generated and/or analyzed during the current study are available from the corresponding author upon reasonable request.
